# Neoadjuvant pembrolizumab-based therapy versus dual HER2 blockade in early breast cancer: comparable surgical complication rates in a real-world cohort

**DOI:** 10.3389/fmed.2026.1917346

**Published:** 2026-09-03

**Authors:** Kerstin Wimmer, Katharina Kantner, Ruth Exner, Rupert Bartsch, Zsuzsanna Bago-Horvath, Stefan Konrad, Florian Fitzal, Maximilian Marhold

**Affiliations:** 1 Department of General Surgery, Division of Visceral Surgery and Comprehensive Cancer Center, Medical University Vienna, Vienna, Austria; 2 Department of Medicine I, Division of Oncology, Medical University Vienna, Vienna, Austria; 3 Department of Pathology, Division of Oncology, Breast Health Center and Comprehensive Cancer Center, Medical University Vienna, Vienna, Austria; 4 Department of Radiation Oncology, Medical University Vienna, Vienna, Austria; 5 Department of General Surgery, Hanusch Hospital, Vienna, Austria

**Keywords:** early breast cancer (EBC), immunotherapy, neoadjuvant chemoimmunotherapy, neoadjuvant systemic therapy, surgical complications

## Abstract

**Background:**

Neoadjuvant systemic therapy (NST) has become a cornerstone in the management of aggressive early breast cancer (BC) subtypes, including triple-negative (eTNBC) and HER2-positive disease. Immune checkpoint inhibition with pembrolizumab represents an active immunotherapeutic approach, whereas dual HER2 blockade with trastuzumab and pertuzumab constitutes passive immunotherapy in early HER2-positive BC. Although both strategies demonstrate substantial antitumor efficacy, real-world data directly comparing their surgical safety profiles are limited.

**Materials and methods:**

This retrospective single-center cohort study included 99 patients with early BC treated with NST between 2014 and 2025. A total of 33 patients with TNBC received pembrolizumab-based therapy according to the KEYNOTE-522 regimen, whereas 66 patients with HER2-positive disease received chemotherapy combined with trastuzumab and pertuzumab. Surgical adverse events within 30 days postoperatively were assessed using the Clavien–Dindo classification and the Comprehensive Complication Index (CCI). Complication rates and severity were compared between groups.

**Results:**

The overall postoperative complication rate was 25.3%, with no significant difference between TNBC and HER2-positive cohorts (27.3% vs. 24.2%, *p =* 0.744). Most complications were minor (Clavien–Dindo ≤II), and rates of major complications (≥IIIa) did not differ between groups (*p =* 0.706). Mean CCI scores were comparable (22.1 vs. 24.4; *p =* 0.630). Seroma formation was the most frequent adverse event in both cohorts. In an exploratory multivariable logistic regression analysis, treatment group was not independently associated with postoperative complications. Pathological complete response rates were 63.6% in TNBC and 45.5% in HER2-positive patients. No clinically relevant delays in adjuvant radiotherapy occurred.

**Conclusion:**

No statistically significant differences in postoperative morbidity were observed between treatment groups. These findings support the feasibility of integrating both neoadjuvant treatment strategies into routine surgical practice; however, confirmation in larger prospective studies is warranted.

## Introduction

Recently, female breast cancer (BC) surpassed lung cancer as the most diagnosed cancer worldwide (1). Despite fundamental advances in early detection and progress in BC treatment modalities, it remains the leading cause of cancer-related mortality among women worldwide (2).

The management of aggressive early BC subtypes – early triple negative (eTNBC) and HER2-positive disease – has been revolutionized by monoclonal antibody-based immunotherapy. In HER2-positive disease, trastuzumab and pertuzumab, both monoclonal antibodies targeting the HER2 receptor directly on tumor cells, have been approved as combined therapy in the neoadjuvant (3), adjuvant (4, 5) as well as in the advanced setting (6). In eTNBC, pembrolizumab, an immune checkpoint inhibitor targeting PD-1 on lymphocytes, enhances antitumor immunity by restoring T-cell activity suppressed through PD-1/PD-L1 signaling. Based on the landmark KEYNOTE-522 trial (7), its addition to neoadjuvant systemic therapy (NST) resulted in significant improvements in pathological complete response, event-free survival (8) and lastly overall survival (9), leading to its approval in the neoadjuvant setting.

Although pembrolizumab, trastuzumab and pertuzumab are all humanized IgG1 antibodies, their mechanisms of action differ fundamentally. Passive immunotherapy refers to externally administered agents that mediate direct antitumor effects, whereas active immunotherapy stimulates endogenous immune responses (10–12). HER2-targeted antibodies are traditionally classified as passive immunotherapy (12, 13). They exert antitumor activity primarily through receptor blockade and antibody-dependent cellular cytotoxicity, mediated mainly by natural killer cells (14), resulting in direct tumor cell lysis without necessarily inducing durable adaptive immunity (10). Trastuzumab inhibits HER2 homodimerization, whereas pertuzumab blocks HER2 heterodimerization, particularly with HER3, thereby providing complementary pathway inhibition (10, 15). In contrast, pembrolizumab represents active immunotherapy: by blocking PD-1 on T cells, it restores tumor-specific adaptive immune responses (16) and may generate long-lasting immunologic memory.

The toxicity profiles of these strategies also differ. HER2-targeted therapy is primarily associated with cardiotoxicity (17–19), whereas checkpoint inhibition leads to immune-related inflammatory adverse events (20–22). Importantly, treatment intensification within each class has not been shown to increase perioperative morbidity. Dual HER2 blockade does not raise postoperative complication rates compared with single-agent therapy, (23) and neoadjuvant chemoimmunotherapy in eTNBC has likewise not been associated with increased surgical complications or delays in surgery or adjuvant radiotherapy (24, 25).

Prospective clinical trials have demonstrated that pembrolizumab and dual HER2 blockade with trastuzumab–pertuzumab (TP), when administered in combination with standard chemotherapy, provide substantial antitumor efficacy with an overall manageable safety profile. Nevertheless, real-world data – particularly with respect to surgical outcomes – remain limited. While immune-related adverse events associated with pembrolizumab have been described in the neoadjuvant setting (20–22, 26), data on surgical adverse events in real-world populations are scarce (24) and direct comparisons between passive immunotherapy (TP) and active immune checkpoint inhibition (pembrolizumab) are lacking.

Although large prospective trials have reported no increase in postoperative complications with neoadjuvant therapy (27–29), skepticism persists, partly driven by heterogeneous older studies (30) and cognitive biases (31). Robust real-world evidence on surgical safety is therefore essential. Accordingly, this study evaluates and compares surgical adverse event rates in patients receiving neoadjuvant chemoimmunotherapy – pembrolizumab for eTNBC and TP for HER2+ early breast cancer.

## Materials and methods

This retrospective single-center cohort study included 99 patients, comprising 66 with histologically confirmed HER2-positive breast cancer and 33 with TNBC, reflecting a planned allocation ratio of approximately 2:1 (HER2-positive:TNBC). All patients received NST at the Department of Oncology, General Hospital of Vienna, between May 2014 and February 2025.

Only patients who had undergone primary tumor resection before data cutoff were included to ensure a minimum postoperative follow-up of 30 days.

The HER2-positive cohort received a tailored treatment including chemotherapy and the monoclonal antibodies trastuzumab and pertuzumab, whilst patients in the TNBC cohort were treated with neoadjuvant chemotherapy in combination with pembrolizumab, according to the KEYNOTE-522 regimen (7). This regimen consisted of 12 weekly cycles of paclitaxel plus carboplatin, followed by 4 cycles of anthracycline-based chemotherapy administered every 3 weeks as epirubicin/cyclophosphamide, in accordance with institutional standards, with pembrolizumab given concurrently throughout treatment. All HER2-positive patients were treated between 2014 and 2018, while TNBC patients received neoadjuvant treatment between 2020 and 2025. Surgical outcomes and pCR-rates were analyzed. In addition to treatment regimen and surgical variables, information on chemotherapy dose reductions, treatment discontinuations, interval between completion of neoadjuvant therapy and surgery, immune-related adverse events, corticosteroid use, and treatment delays was extracted from the electronic medical records where available. Surgical adverse events within the first 30 postoperative days were graded according to the Clavien-Dindo Classification (32) and the Comprehensive Complication Index (CCI) (33) was employed to assess overall postoperative morbidity. Differences in complication severity measured by the Clavien-Dindo Classification between TNBC and HER2-positive groups were assessed using the Mann-Whitney U test. A sensitivity analysis comparing rates of major (grade ≥ III) complications was performed using the Chi-square test. Surgical complications were compared between patients of the TNBC and HER2-positive cohort using two-sample non-parametric tests. Continuous variables were described using medians and interquartile range (IQR) or mean ± standard deviation (SD), as appropriate, whereas categorical variables were described using counts and percentages. Categorical variables, including complication grades, were compared between the TNBC and the HER2-positive groups using the Pearson Chi-square test. When more than 20% of the expected cell counts were below 5, Fisher’s exact test was applied instead to ensure validity of the results. To evaluate whether implant-based breast reconstruction (IBBR), including either direct-to-implant reconstruction or expander placement, was associated with increased postoperative morbidity, subgroup analyses were performed separately within the TNBC and HER2-positive cohorts. Within each treatment group, complication rates were compared between patients undergoing IBBR and those without reconstruction. To account for potential confounding factors, an exploratory multivariable logistic regression analysis was performed with postoperative complication (yes/no) as the dependent variable. Based on clinical relevance and the limited number of postoperative events, age, body mass index (BMI), treatment group (TNBC versus HER2-positive disease), axillary lymph node dissection (ALND), and IBBR were included as independent variables. Odds ratios (ORs) with 95% confidence intervals (CIs) were calculated.

All analyses were two-tailed and a *p*-value of < 0.05 was considered statistically significant. IBM SPSS Statistics Version 29.0 was used for all analyses. Figures were generated using Graph Pad Prism7.0a for Macintosh. The study was approved by the Ethics Committee of the Medical University of Vienna (vote 1705/2025). As this was a retrospective analysis, the Ethics Committee waived the requirement for written informed patient consent.

## Results

A total of 99 patients who received NST at the Department of Oncology at the General Hospital of Vienna between May 2014 and February 2025 were included. Of these, 33 patients were diagnosed with TNBC and 66 patients had HER2-positive breast cancer.

In the TNBC cohort (*N =* 33, all female), 51.5% of patients (*n =* 17) were premenopausal, 27.3% (*n =* 9) were perimenopausal and 21.2% (*n =* 7) were postmenopausal. The mean age was 45.8 ± 12.4 years (SD), and the mean BMI was 25 ± 4.3 kg/m^2^. In the HER2-positive cohort (*N =* 66), 65.2% (*n =* 43) had triple-positive disease, while 34.8% (*n =* 23) had HR-negative/HER2-positive disease. One male patient was included in this cohort; the remaining 65 patients were female. Among the female patients, 28.8% (*n =* 19) were premenopausal, 57.6% (*n =* 38) perimenopausal, and 7.6% (*n =* 5) postmenopausal. For four patients, menopausal status was not documented. The mean age was 53.6 ± 11.5 years (SD), and the mean BMI was 25.7 ± 5.4 kg/m^2^. Compared with the HER2-positive cohort, patients with TNBC were significantly younger (*p =* 0.002), more frequently premenopausal (*p =* 0.005), and had higher tumor grade (*p* < 0.001) and Ki-67 proliferation indices (p < 0.001). Clinicopathological characteristics at diagnosis are summarized in Table 1.

**TABLE 1:** Clinicopathological characteristics for patients of the TNBC and the HER2-positive cohort.

Clinicopathological characteristic	TNBC cohort	HER2-positive cohort	*p*-values^§^
	(*N =* 33)	(*N =* 66)	
Hormone receptor status			
HR+		43 (65.2%)	
HR−		23 (34.8%)	
Age			
Mean (SD)	45.8 (12.4)	53.6 (11.5)	0.002
Gender			1.000
Female	33 (100%)	65 (98.5%)	
Male	0 (0%)	1 (1.5%)	
BMI			
Mean (SD)	25 (4.3)	25.7 (5.4)	0.557
Menopausal status			0.005
Premenopausal	17 (51.5%)	19 (28.8%)	
Perimenopausal	9 (27.3%)	38 (57.6%)	
Postmenopausal	7 (21.2%)	5 (7.6%)	
Missing	0 (0%)	4 (6.1%)	
cT-stage			0.557
T0	1 (3%)	0 (0%)	
T1	11 (33.3%)	21 (31.8%)	
T2	16 (48.5%)	36 (54.5%)	
T3	5 (15.2%)	6 (9.1%)	
T4	0 (0%)	1 (1.5%)	
Missing	0 (0%)	2 (3%)	
N-stage			0.83
0	11 (33.3%)	37 (56.1%)	
1	14 (42.4%)	24 (36.4%)	
2	6 (18.2%)	4 (6.1%)	
3	1 (3%)	1 (1.5%)	
Missing	1 (3%)	0 (0%)	
Grade			<0.001
G1	0 (0%)	1 (1.5%)	
G2	0 (0%)	16 (24.2%)	
G3	30 (90.9%)	44 (66.7%)	
Missing	3 (9.1%)	5 (7.6%)	
MIB-1/Ki-67 (%)			
Mean (SD)	80.2 (13.8)	48.9 (18.7)	<0.001
BRCA mutation status			0.624
Wildtype	23 (69.7%)	6 (9.1%)	
BRCA1 mutant	5 (15.2%)	0 (0%)	
BRCA2 mutant	3 (9.1%)	1 (1.5%)	
Missing	2 (6.1%)	59 (89.4%)	

TNBC, triple negative breast cancer, HER2, human epidermal growth factor receptor 2; HR, hormone receptor; +, positive; −, negative; SD, standard deviation; BMI, body mass index; MIB-1, MIB-1 proliferation index. ^§^ *P*-values compare the TNBC cohort with the HER2-positive cohort. *P*-values of < 0.05 were considered as statistically significant.

In the TNBC cohort, all 33 patients received neoadjuvant pembrolizumab. The chemotherapy backbone was uniform and consistent with the KEYNOTE-522 regimen, consisting of 12 weekly cycles of carboplatin plus paclitaxel followed by 4 cycles of anthracycline-based chemotherapy with epirubicin in combination with cyclophosphamide. In the HER2-positive cohort, the majority (*n =* 54; 81.8%) received dual HER2 blockade in combination with anthracycline-based chemotherapy, while 7 patients (10.6%) underwent an anthracycline-free regimen. NST containing vinorelbine monotherapy was administered in four patients (6.1%), and one patient received TP without chemotherapy because of comorbidities (1.5%). Treatment delivery differed between the two cohorts. Chemotherapy dose reductions were documented in 27 TNBC patients (81.8%) compared with 13 HER2-positive patients (19.7%). Chemotherapy was discontinued in three TNBC patients (9.1%) and eight HER2-positive patients (12.1%), whereas antibody treatment was discontinued in five patients in each cohort (15.2% vs. 7.6%). The interval between completion of NST and surgery was similar between the two cohorts, with a median of 4.0 weeks (IQR, 3.7–4.9) in the TNBC cohort and 3.9 weeks (IQR, 3.0–5.1) in the HER2-positive cohort, indicating no clinically relevant difference in surgical timing. In the TNBC cohort, immune-related adverse events occurred in 12 of 33 patients (36.4%), with corticosteroid treatment required in 9 patients (75%). No clinically relevant delays in surgery attributable to immune-related adverse events (irAEs) were observed. Among patients who developed irAEs, the median interval between completion of neoadjuvant systemic therapy and surgery was 4.1 weeks (IQR, 3.7–5.1). The spectrum of irAEs included endocrine (hypothyroidism), dermatologic (autoimmune dermatitis, erythema multiforme), gastrointestinal/hepatic (type A gastritis, autoimmune hepatitis), pulmonary (pneumonitis/interstitial lung disease), musculoskeletal (arthralgia/joint effusions), cardiovascular (pericardial effusion, myocarditis/coronary artery dissection), and hematologic (eosinophilia) manifestations. Pembrolizumab was discontinued in four of the 12 patients (33.3%) who experienced irAEs because of treatment-related toxicity.

**FIGURE 1:**
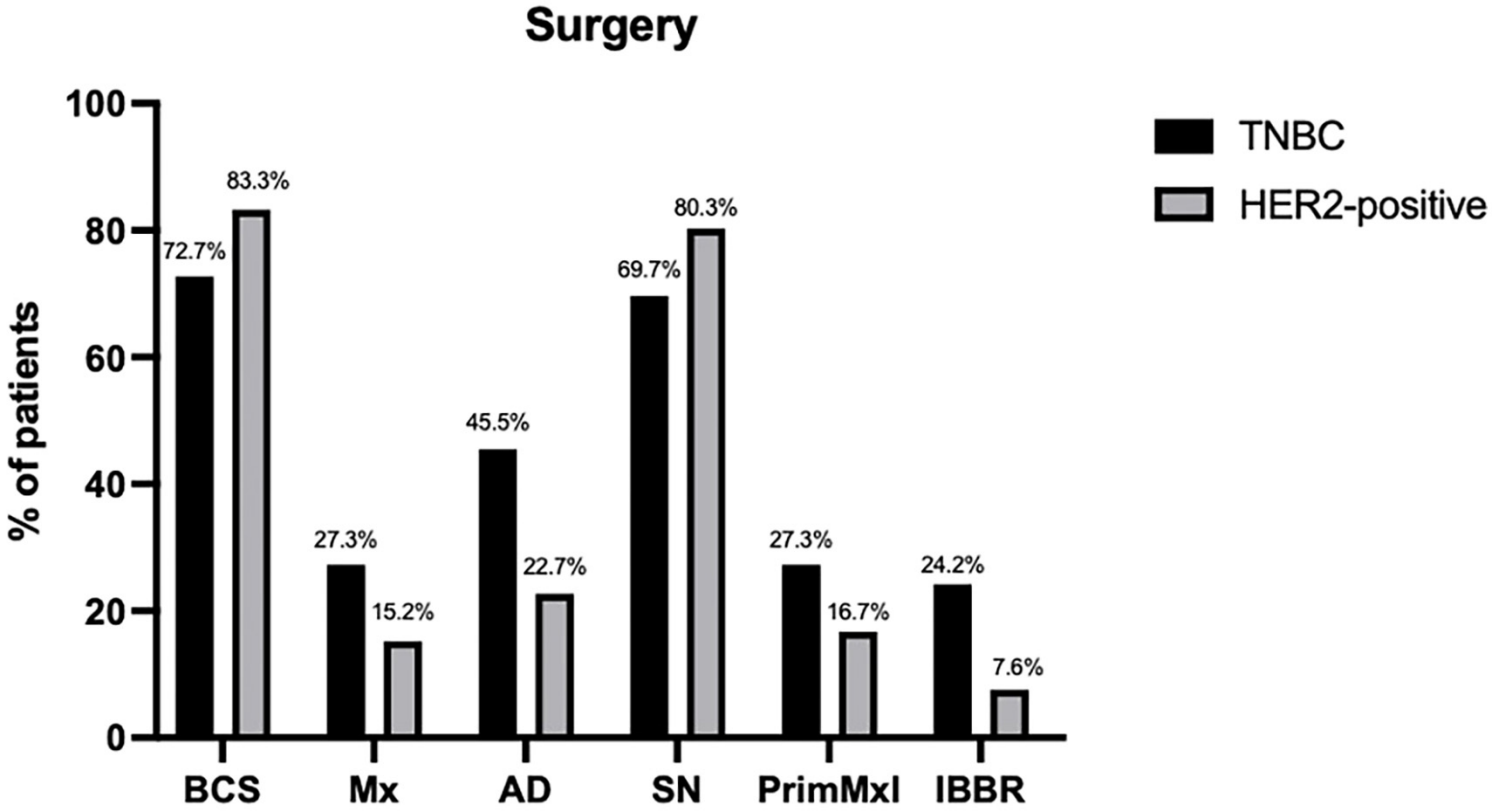
Surgical procedures performed in the TNBC cohort (*N =* 33) and the HER2-positive cohort (*N =* 66), presented as percentages. BCS, Breast-conserving surgery; Mx, mastectomy; AD, axillary lymph node dissection; SN, sentinel lymph node; PrimMxI, primary mastectomy indication; IBBR, implant-based breast reconstruction.

Surgical therapies did not differ substantially between subgroups. Breast-conserving surgery was performed in 72.7% of TNBC patients (*n =* 24) and 83.3% of HER2-positive patients (*n =* 55). Mastectomy was carried out in 27.3% of TNBC patients (*n =* 9) and 15.2% of HER2-positive patients (*n =* 10). Regarding axillary surgery, ALND was performed in 15 TNBC patients (45.5%) and in 15 HER2-positive patients (22.7%). Sentinel lymph node biopsy was conducted in 23 TNBC patients (69.7%) and 53 HER2-positive patients (80.3%). IBBR was performed in 13 patients overall (13.1%), comprising 8 patients in the TNBC cohort (24.2%) and 5 patients in the HER2-positive cohort (7.6%). Figure 1 provides an overview of the surgical interventions applied after neoadjuvant chemoimmunotherapy.

The pCR rate was 63.6% (*n =* 21) in the TNBC cohort and 45.5% (*n =* 30) in the HER2-positive subgroup. In the TNBC cohort, 22 patients (66.7%) achieved RCB 0, two patients achieved RCB I (6.1%) and five patients (15.2%) RCB II; RCB data were missing for four patients. In the HER2-positive cohort, 32 patients (48.5%) achieved RCB 0, 11 patients (16.7%) RCB I, and 12 patients (18.2%) RCB II; RCB was missing for 11 patients. Information on treatment modalities and tumor response to treatment are shown in Table 2.

**TABLE 2:** Neoadjuvant chemotherapy regimens, treatment characteristics, and tumor response in the TNBC and HER2-positive cohorts.

Variable	TNBC cohort	HER2-positive cohort
	(*N =* 33)	(*N =* 66)
Weeks of neoadjuvant treatment		
Mean (SD)	22.0 (4.5)	28.7 (17.4)
Chemotherapy according to KN-522 for TNBC cohort		
	33 (100%)	
Chemotherapy regimen in HER2-positive cohort		
NST containing anthracyclines		54 (81.8%)
NST without anthracyclines		7 (10.6%)
NST containing vinorelbine		4 (6.1%)
Chemotherapy-free regimen		1 (1.5%)
Deviation of chemotherapy regimen, n (%)		
Dose reduction of chemotherapy	27 (81.8%)	13 (19.7%)
Discontinuation of chemotherapy	3 (9.1%)	8 (12.1%)
Discontinuation of of antibody treatment	5 (15.2%)	5 (7.6%)
Cycles of Pembrolizumab		
Mean (SD)	7.2 (1.7)	
Tumor response to NST (%)		
Pathological complete response	21 (63.6%)	30 (45.5%)
RCB, n (%)		
0	22 (66.7%)	32 (48.5%)
I	2 (6.1%)	11 (16.7%)
II	5 (15.2%)	12 (18.2%)
III	0 (0%)	0 (0%)
Missing	4 (12.1%)	11 (16.7%)

TNBC, triple negative breast cancer, HER2, human epidermal growth factor receptor 2; SD, standard deviation; KN-522, Keynote-522 trial; NST, neoadjuvant systemic therapy; RCB, Residual cancer burden.

### Surgical complication rate and Clavien-Dindo score within the first 30 days after surgery

The overall complication rate in the entire study cohort was 25.3%, with no statistically significant difference between TNBC (*n =* 9; 27.3%) and HER2-positive patients (*n =* 16; 24.2%, *p =* 0.744). Seroma formation was the most frequent adverse event (AE) in both subgroups. Postoperative hemorrhage occurred in one patient in each subgroup. Additional complications occurred exclusively in the HER2-positive group, including wound-healing complications in three patients and postoperative infections in three patients (Table 3).

**TABLE 3:** Rates of surgical complications and corresponding interventions in patients with TNBC and HER2-positive breast cancer.

Variable	All patients	TNBC cohort	HER2-positive cohort	*p*-values^§^
	(*N =* 99)	(*N =* 33)	(*N =* 66)	
Postoperative surgical complications, n (%)				
Any complication	25 (25.3%)	9 (27.3%)	16 (24.2%)	0.744
Postoperative hemorrhage	2 (2%)	1 (3%)	1 (1.5%)	1.000
Wound healing deficit	5 (5.1%)	2 (6.1%)	3 (4.5%)	1.000
Infection	3 (3%)	0 (0%)	3 (4.5%)	0.549
Seroma	22 (22.2%)	8 (24.2%)	14 (21.2%)	0.732
Multiple complications	5 (5.1%)	1 (3%)	4 (6.1%)	0.662
Intervention for surgical complication, n (%)				
Treatment required	20 (20.2%)	7 (21.2%) *	14 (21.2%)	1.000
AE requiring outpatient management	17 (17.2%)	6 (18.2%)	11 (16.7%)	0.789
AE requiring inpatient management	4 (4%)	1 (3%)	3 (4.5%)	1.000
Seroma puncture				
Any	14 (14.1%)	4 (12.1%)	10 (15.2%)	1.000
Once	8 (8.1%)	2 (6.1%)	6 (9.1%)	1.000
More than once	6 (6.1%)	2 (6.1%)	4 (6.1%)	1.000
Antibiotics	7 (7.1%)	1 (3%)	6 (9.1%)	0.419
Radiotherapy postponed due to AE	1 (1%)	0 (0%)	1 (1.5%)	1.000
Clavien-Dindo Classification, n (%)				
0	74 (74.7%)	24 (72.7%)	50 (75.8%)	
I	6 (6.1%)	3 (9.1%)	3 (4.5%)	
II	1 (1%)	0 (0%)	1 (1.5%)	
IIIa	15 (15.2%)	4 (12.1%)	11 (16.7%)	
IIIb	2 (2%)	1 (3%)	1 (1.5%)	
IV	0 (0%)	0 (0%)	0 (0%)	
V	0 (0%)	0 (0%)	0 (0%)	
Missing	1 (1%)	1 (3%)	0 (0%)	
Minor complication (grade I + II)	7 (7.1%)	3 (9.1%)	4 (6.1%)	0.683
Major complication (grade ≥ III)	17 (17.2%)	5 (15.2%)	12 (18.2%)	0.706
CCI-score				
Mean (SD)	23.5 (9.6)	22.1 (11.7)	24.4 (8.2)	0.630
Median	26.2	26.2	26.2	0.716

TNBC, triple negative breast cancer, HER2, human epidermal growth factor receptor 2; AE, adverse event; CCI, comprehensive complication index. ^§^ *P*-values compare the TNBC cohort with the HER2-positive cohort. *P*-values of < 0.05 were considered as statistically significant. *Information for one patient missing.

In both the TNBC and HER2-positive cohorts, rates of wound healing deficits, postoperative hemorrhage, surgical site infection, and seroma formation were comparable between patients with and without IBBR (Supplementary Table 1). No increase in minor or major complication rates was observed in association with IBBR within either treatment cohort.

To account for potential confounding factors, an exploratory multivariable logistic regression analysis including age, BMI, treatment group, ALND, and IBBR was performed (Table 4). Treatment group was not significantly associated with postoperative complications (OR 1.13, 95% CI 0.39–3.25; *p =* 0.826). In contrast, ALND was independently associated with an increased risk of postoperative complications (OR 3.36, 95% CI 1.23–9.18; *p =* 0.018), whereas age, BMI, and immediate breast reconstruction were not significant predictors.

**TABLE 4:** Exploratory multivariable logistic regression analysis of factors associated with postoperative complications.

Variable	Odds Ratio (OR)	95% confidence interval	*p*-value
Variable			
HER2-positive vs. TNBC	1.13	0.39–3.25	0.826
Age (per year)	1.00	0.96–1.04	0.819
BMI (per kg/m^2^)	0.99	0.89–1.09	0.792
Axillary lymph node dissection (ALND)	3.36	1.23–9.18	0.018
Immediate breast reconstruction (IBBR)	0.14	0.01–1.28	0.081

Postoperative complication (yes/no) was used as the dependent variable. The model included age, BMI, treatment group (HER2-positive disease as the reference category), axillary lymph node dissection, and immediate breast reconstruction. BMI, body mass index; IBBR, immediate breast reconstruction; TNBC, triple-negative breast cancer.

**FIGURE 2:**
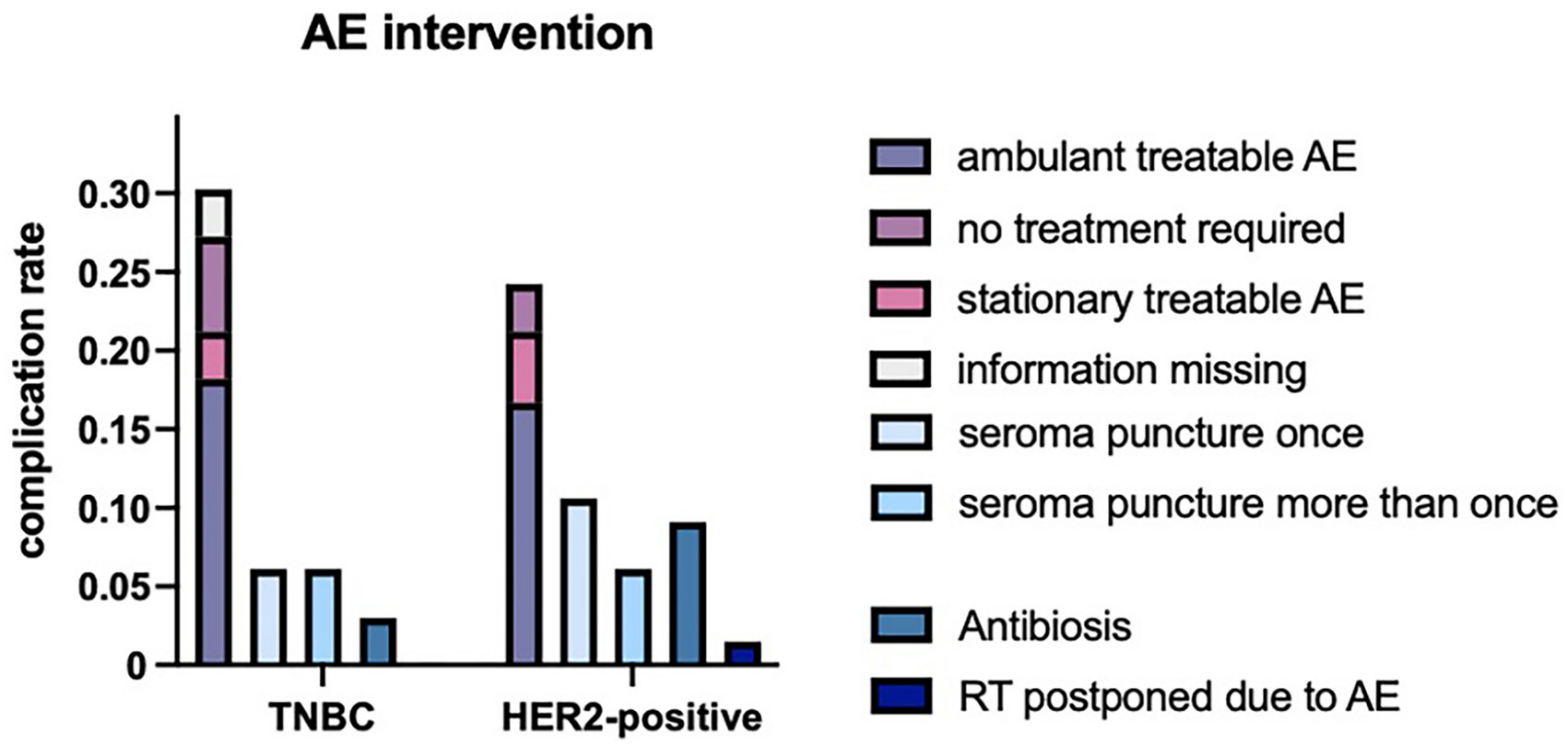
Interventions for surgical complications in the TNBC (*n =* 9) and HER2-positive (*n =* 16) cohorts. Percentages are calculated relative to the number of patients experiencing postoperative surgical complications. Data on seroma puncture refer to the number of patients developing a seroma (TNBC: *n =* 9; HER2+: *n =* 16). For one patient in the TNBC group this information was missing. TNBC, triple negative breast cancer, HER2, human epidermal growth factor receptor 2; AE, adverse event; RT, radiotherapy.

**FIGURE 3:**
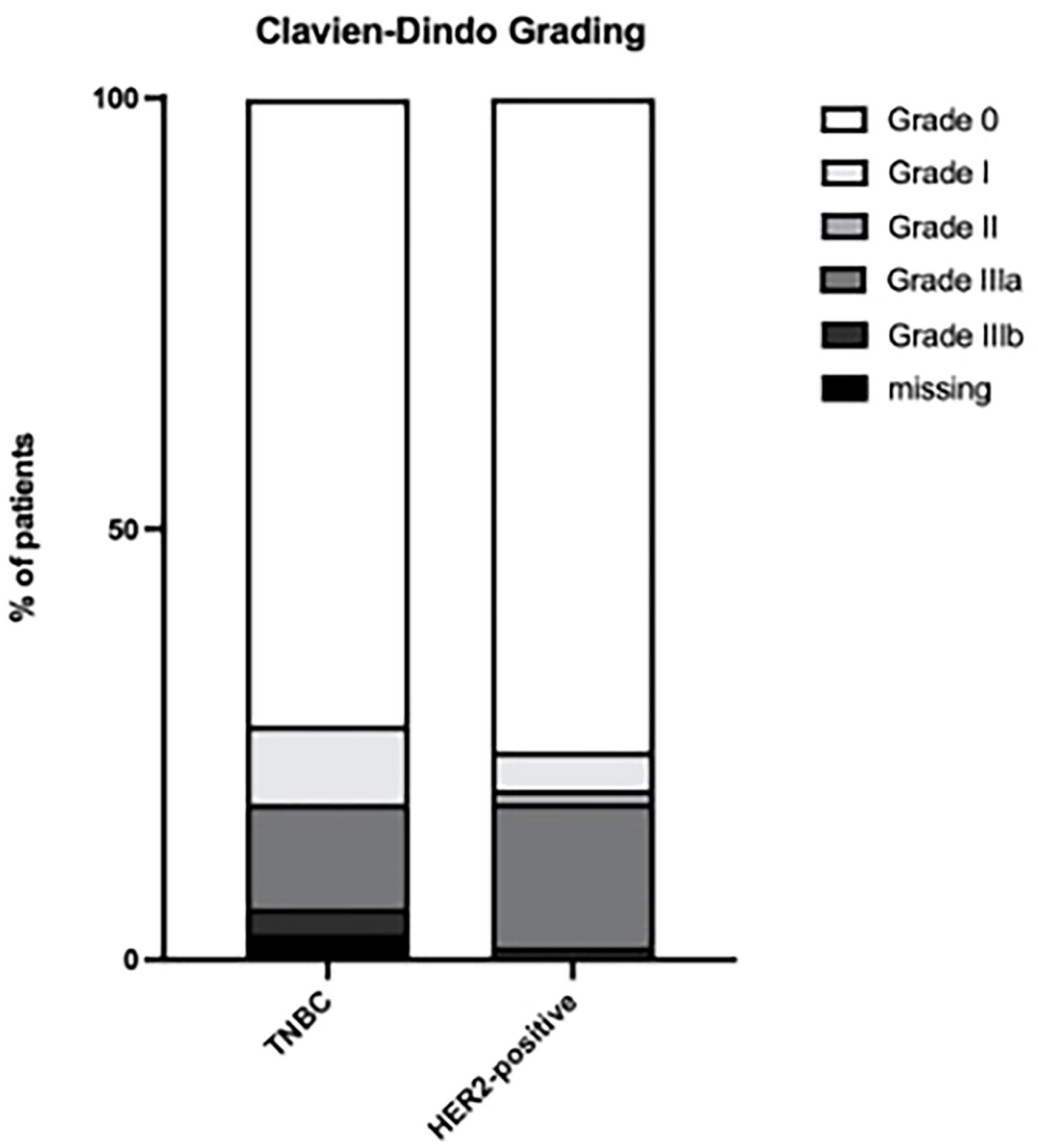
Clavien-Dindo classification for patients of the TNBC and the HER2-positive cohort. Percentages are calculated relative to the number of patients experiencing postoperative surgical complications. TNBC, triple negative breast cancer, HER2, human epidermal growth factor receptor 2.

Figure 2 provides an overview of the interventions required for the management of postoperative complications. The Clavien–Dindo classification of all adverse events is shown in Figure 3. There was no difference in the distribution of minor (Clavien–Dindo ≤ II) or major complications (Clavien–Dindo ≥ IIIa) between the two treatment groups (minor complications: *p =* 0.683; major complications: *p =* 0.706; Table 3). The mean CCI in the TNBC cohort was 22.1 (SD 11.7), compared with 24.4 (SD 8.2) in the HER2-positive cohort, again without statistically significant differences (*p =* 0.630; Table 3). Mean Comprehensive Complication Index (CCI) scores did not differ between TNBC patients with and without IBBR. Similarly, no difference in mean CCI was observed between HER2-positive patients undergoing IBBR and those without reconstruction.

Similarly, no statistically significant group differences were observed regarding the type of intervention required for postoperative complications. There were no differences in the rates of outpatient versus inpatient management of AEs, nor in the frequency of seroma punctation. The rates of prescribed oral antibiotics also did not differ between TNBC and HER2-positive patients (3% vs. 9.1%; *p =* 0.419). Postponement of radiotherapy due to surgical complications was necessary in one patient in the HER2-positive cohort.

During a mean follow-up period of 3.9 years ± 2.9 years, re-surgery was required in three TNBC patients (9.1%) and in 11 HER2-positive patients (16.7%). The most common indication for reoperation was locoregional tumor relapse (*n =* 9), followed by expander exchange or implant-related procedures (*n =* 4). Positive surgical margins resulted in reoperation in three cases (one in the TNBC and two in the HER2-positivesubgroup).

## Discussion

The principal finding of this retrospective single-center study is that NST including active (checkpoint inhibitor for eTNBC) or passive (dual monoclonal antibody treatment against HER2 for early HER2-positive BC) immunotherapy did not translate into differential surgical morbidity. No statistically significant difference of surgical complications was observed between TNBC (27.3%) and HER2-positive patients (24.2%, *p =* 0.744), indicating that subtype-specific treatment intensity did not compromise surgical safety. To our knowledge, this is the first study to report such a finding. Previous studies have shown that neoadjuvant therapy – including chemo-immunotherapy and HER2-directed approaches – did not significantly increase postoperative complication risk when standard surgical techniques are applied (24, 34, 35), but no direct comparisons between active and passive immunotherapies were performed in the studies.

With regard to surgical complication rates, recently published data from the randomized KEYNOTE-522 trial suggest that the addition of immune checkpoint inhibition to neoadjuvant chemotherapy does not increase postoperative morbidity (36). Similar findings have been reported in real-world settings, where pembrolizumab was not associated with higher rates of surgical site infection, seroma or hematoma formation, reoperation within 30 days after surgery, or delays in adjuvant therapy (24, 25, 37).

The interpretation of our findings should be considered in the context of differences in both baseline characteristics and surgical management between the two cohorts. Patients with TNBC tended to undergo more extensive breast and axillary surgery, including higher rates of mastectomy and ALND. As the extent of breast and axillary surgery is a well-established determinant of postoperative morbidity (38), these differences may have influenced postoperative outcomes. In addition, the TNBC cohort differed from the HER2-positive cohort with respect to several baseline characteristics, including younger age, menopausal status, tumor grade, and Ki-67 proliferation index, reflecting the distinct biological characteristics of these disease entities. Nevertheless, postoperative complication rates remained comparable between the groups. Moreover, exploratory multivariable logistic regression analysis adjusting for age, BMI, ALND, and IBBR did not identify treatment group as an independent predictor of postoperative complications, whereas ALND emerged as an independent predictor of postoperative complications.

Systemic treatment tolerability, however, differed more noticeably between groups. Dose reductions were more common in TNBC (81.8%) than in HER2-positive patients (19.7%), and chemotherapy discontinuation occurred in 9.1% and 12.1%, respectively. These real-world treatment modification rates, particularly the high frequency of dose reductions in the TNBC cohort, exceed those reported in pivotal trials, such as KEYNOTE-522 (7) and major HER2 trials (39–41), where stringent eligibility criteria, close monitoring, and protocolized supportive care minimize unplanned dose alterations. The higher modification rates observed in our cohort likely reflect the greater clinical heterogeneity and differences in real-world toxicity management compared with trial populations, as previously discussed by our group (26). Treatment-related toxicity represents another clinically relevant factor that may influence perioperative outcomes following neoadjuvant therapy. In our cohort, information on immune-related adverse events (irAEs), corticosteroid use, treatment delays, and treatment modifications was routinely collected. We have previously demonstrated that the occurrence of irAEs during neoadjuvant pembrolizumab therapy was associated with improved pathological complete response (26), a finding that has recently been confirmed in an independent multicenter cohort by Güren et al. (42). Together, these observations suggest that irAEs may represent clinically relevant biomarkers of immune activation and treatment efficacy. However, the primary objective of the present study was to evaluate postoperative surgical morbidity rather than the relationship between treatment-related toxicity and surgical outcomes. Although treatment modifications, chemotherapy discontinuations, and antibody discontinuations were captured, the limited number of postoperative events precluded robust analyses assessing the independent impact of specific toxicities, corticosteroid exposure, or treatment delays on postoperative morbidity. Larger studies with sufficient event numbers are needed to determine whether these treatment-related factors influence surgical outcomes.

Despite these treatment modifications, pCR outcomes remained robust in our cohort. The TNBC cohort achieved a pCR rate of 63.6%, closely approximating the 64.8% reported in KEYNOTE-522 (7). This suggests that meaningful tumor eradication is achievable even when frequent chemotherapy dose reductions are required. The HER2-positive pCR rate of 45.5% is comparable with the 45.8% observed in the docetaxel/trastuzumab/pertuzumab arm in NeoSphere (40), but somewhat lower than the 55–65% observed with dual HER2 blockade plus chemotherapy including carboplatin and/or an anthracycline in trials such TRYPHAENA (57.3–66.2%) (39), and TRAIN-2 (67–68%) (41). Variation in real-world chemotherapy dose intensity – known to influence pCR (43, 44) – together with patient heterogeneity and modest discontinuation rates may account for these differences.

This retrospective, single-center study is subject to inherent limitations, including potential selection bias and limited generalizability. The limited sample size may have been insufficient to detect small differences in postoperative risk, and the occurrence of rare complications could not be comprehensively assessed. These limitations are particularly relevant to subgroup analyses stratified by BC subtype and IBBR. Although no statistically significant differences in surgical morbidity were observed between patients receiving neoadjuvant pembrolizumab-based therapy and those treated with dual HER2 blockade, these findings should be interpreted with caution and require validation in larger prospective multicenter cohorts. Although an exploratory multivariable logistic regression analysis was performed to adjust for clinically relevant confounders, the limited number of postoperative events restricted the complexity of the model and may have limited the ability to detect smaller associations. Therefore, residual confounding cannot be excluded. Larger multicenter cohorts will be required to validate these findings and permit more comprehensive adjustment for clinical and treatment-related confounders. Furthermore, the extended study period as well as temporal differences between the cohorts should be considered, as all patients with HER2-positive disease were treated before 2019, whereas the TNBC cohort received neoadjuvant therapy after 2020, potentially introducing further treatment- and time-related bias.

In conclusion, in this retrospective real-world cohort, no statistically significant differences in postoperative morbidity were observed between patients receiving neoadjuvant pembrolizumab-based therapy and those treated with dual HER2 blockade. These findings support the feasibility of integrating contemporary neoadjuvant immunotherapeutic strategies for eTNBC and HER2-positive BC into routine surgical practice. However, given the retrospective design, inherent differences between the study cohorts, and limited sample size, confirmation in larger prospective multicenter studies is warranted. Although chemotherapy modifications were more frequent – particularly in TNBC – pCR rates remained within the expected ranges of pivotal clinical trials. Overall, the present data highlight the importance of optimized supportive care to preserve treatment intensity while minimizing treatment-related morbidity and maintaining favorable surgical outcomes.

## Data Availability

The raw data supporting the conclusions of this article will be made available by the authors, without undue reservation.
