# Supplementary material for: Neoadjuvant pembrolizumab-based therapy versus dual HER2 blockade in early breast cancer: comparable surgical complication rates in a real-world cohort
Source: Front Med (Lausanne). 2026 Sep 3;13:1917346. doi: 10.3389/fmed.2026.1917346 (PMC13581936; doi:10.3389/fmed.2026.1917346)
Supplement: Supplementary file 1 [file Table_1.docx]

**Supplementary tables**

Supplementary Table S1. Postoperative surgical complications, Clavien-Dindo Classification and reported CCI-scores, in TNBC and HER2-positive patients with or without implant-based breast reconstruction.

Abbreviations: TNBC, triple negative breast cancer, HER2, human epidermal growth factor receptor 2; IBBR, implant-based breast reconstruction; CCI, Comprehensive Complication Index.

|  | TNBC cohort  (N =33) | | p-values^§^ | HER2-positive cohort  (N = 66) | | p-values^§^ |
| --- | --- | --- | --- | --- | --- | --- |
|  | Non-IBBR  (n=25) | IBBR  (n=8) |  | Non-IBBR  (n=61) | IBBR  (n=5) |  |
| Postoperative surgical complications, n (%) | | | | | | |
| Postoperative hemorrhage | 1 | 0 | 1.000 | 1 | 0 | 1.000 |
| Wound healing deficit | 2 | 0 | 1.000 | 3 | 0 | 1.000 |
| Infection | 0 | 0 | nd | 3 | 0 | 1.000 |
| Seroma | 8 | 0 | .152 | 13 | 1 | 1.000 |
| Clavien-Dindo Classification, n (%) | | | | | | |
| 0 | 16 | 8 |  | 46 | 4 |  |
| I | 3 | 0 |  | 3 | 0 |  |
| II | 0 | 0 |  | 1 | 0 |  |
| IIIa | 4 | 0 |  | 10 | 1 |  |
| IIIb | 1 | 0 |  | 1 | 0 |  |
| IV | 0 | 0 |  | 0 | 0 |  |
| V | 0 | 0 |  | 0 | 0 |  |
| missing |  |  |  |  |  |  |
| Major complications (grade I + II) | 5 | 0 | 0.302 | 11 | 1 | 1.000 |
| Minor complications (grade ≥ III) | 3 | 0 | 0.560 | 0 | 4 | 1.000 |
| CCI-score | | | | | | |
| Mean (SD) | 22.1 (11.7) | 0 | 0.905 | 23.9 (8.9) | 5.5 (12.3) | 0.905 |
| Median | 26.2 | 0 |  | 26.2 | 0 |  |

^§^P-values compare the TNBC cohort with the HER2-positive cohort. P-values of <0.05 were considered as statistically significant.
